# Usefulness of 18F-Fluorodeoxyglucose Positron Emission Tomography in Diagnosing Polymyalgia Rheumatica and Large-Vessel Vasculitis: A Case-Control Study

**DOI:** 10.3390/jcm12082844

**Published:** 2023-04-13

**Authors:** Anne Desvages, Florent Hives, Xavier Deprez, Adeline Pierache, Hélène Béhal, René-Marc Flipo, Julien Paccou

**Affiliations:** 1 Department of Rheumatology, Lille University Hospital, University of Lille, CHU Lille, 59000 Lille, France; 2 Department of Nuclear Medicine, Lille University Hospital, University of Lille, CHU Lille, 59000 Lille, France; 3 Department of Rheumatology, Valenciennes Hospital, 59300 Valenciennes, France; 4 METRICS: Evaluation des Technologies de Santé et des Pratiques Médicales, University of Lille, CHU Lille, ULR 2694, 59000 Lille, France

**Keywords:** polymyalgia rheumatica, FDG-PET/CT, large-vessel vasculitis, giant cell arteritis

## Abstract

Introduction: We aimed to evaluate the utility of FDG-PET/CT in diagnosing polymyalgia rheumatica (PMR) and associated large-vessel vasculitis (LVV). Methods: We analyzed FDG-PET/CT completed between 2015 and 2019 on patients diagnosed with PMR. For comparisons, patients with PMR were matched 1:1 to controls based on age and gender. FDG-PET/CT had been completed on the controls over the same period. The FDG uptake was scored visually for 17 articular or periarticular sites and 13 vascular sites using a semi-quantitative scoring system (score of 0–3). Results: Eighty-one patients with PMR and eighty-one controls were included (mean age 70.7 (9.8) years; 44.4% women). Significant differences between the PMR and control groups were found at all articular and periarticular sites for the following: (i) the FDG uptake score (*p* < 0.001 for all locations); (ii) the number of patients per site with significant FDG uptake (score ≥ 2); (iii) the global FDG articular uptake scores (31 [IQR, 21 to 37] versus 6 [IQR, 3 to 10], *p* < 0.001); and (iv) the number of sites with significant FDG uptake (score ≥ 2) (scores of 0–17) (11 [IQR, 7 to 13] versus 1 [IQR, 0 to 2], *p* < 0.001). No significant differences in the global FDG vascular uptake scores were found between the patients who were considered isolated PMR and the control groups. Conclusions: The FDG uptake score and the number of sites with significant FDG uptake could be pertinent criteria for the diagnosis of PMR. Unlike others, we did not confirm the presence of vascular involvement in patients with isolated PMR.

## 1. Introduction

Polymyalgia rheumatica (PMR) is a relatively common inflammatory rheumatic disease in people who are 50 or older [1]. It is characterized by the occurrence of arthromyalgia of the scapular and pelvic girdles [2], and it is often associated with constitutional symptoms [3,4]. Peripheral joint manifestations are observed in 23–39% of patients [5,6]. In most cases, there is an increase in acute phase reactants [7]. PMR occurs most often in isolation, but there is evidence suggesting that it might be associated with giant cell arteritis (GCA) in 16–21% of cases [7]. Generally, its clinical presentation and biological profile are typical, allowing the initiation of treatment by oral corticosteroid therapy, which is the mainstay of PMR treatment [8]. Therapeutic alternatives have been evaluated, including tocilizumab [9].

There is no gold standard for diagnosing PMR, but classification criteria were jointly published by the European League Against Rheumatism (EULAR) and the American College of Rheumatology (ACR) in 2012, suggesting for the first time the use of imaging support, with the possible use of ultrasound [10]. None of the most common imaging techniques can provide diagnostic certainty of PMR, but, more recently, the use of 18F-fluorodeoxyglucose positron emission tomography/computed tomography (FDG-PET/CT) in the management of PMR, especially for diagnoses and therapeutic monitoring, has been evaluated [11,12,13]. However, the 2012 ACR/EULAR classification criteria do not include the use of this imaging technique [10]. Additionally, some studies have reported non-negligible vascular hyperfixation rates—referred to as associated large-vessel vasculitis (LVV) (11–60.7%)—in patients with PMR [12,14,15] or the possibility of an underlying neoplasia in the setting of paraneoplastic PMR [16,17].

However, studies evaluating the utility of FDG-PET/CT in diagnosing PMR and associated LVV are few and have only been conducted on a small number of patients. All of these findings suggest that FDG-PET/CT imaging may be useful in the management of PMR patients, even though this is still debated. The aim of this study was to evaluate the utility of FDG-PET/CT in diagnosing PMR and silent underlying LVV.

## 2. Patients and Methods

### 2.1. Study Design and Patient Recruitment

This is a multicenter, retrospective study with 2 participating centers (Lille University Hospital and Valenciennes Hospital). We analyzed FDG-PET/CT scans completed between January 2015 and December 2019 on patients diagnosed with PMR admitted to conventional or day hospital units. Patients were hospitalized either for diagnoses or reassessments of active PMR. For the purpose of this study, analyses were not restricted to patients fulfilling the 2012 EULAR/ACR classification criteria for PMR [9]. Patients with a clinical diagnosis of PMR based on an expert opinion were also included in the study, provided they had had at least one FDG-PET/CT scan. 

For comparisons, patients with PMR were matched 1:1 to controls based on age and gender. In patients in the control group, FDG-PET/CT scans had been performed over the same period at Lille University Hospital. Controls had recently suffered a stroke and a scan was performed to determine whether the stroke might be associated with some form of cancer. Exclusion criteria for controls were prior history of inflammatory rheumatic diseases (IRD) and/or vasculitides, including GCA.

The study protocol was accepted by the Lille University Hospital Review Board (n° DEC2019-133), and the study procedures acted in accordance with the Helsinki Declaration of 1975, as revised in 2000. Since this is a retrospective study, it did not require ethical committee approval and complied with French laws and regulations. Informed consent was obtained from all patients for the FDG-PET/CT scans.

### 2.2. Study Protocol

#### 2.2.1. Patient Disease Assessment

Information was obtained by means of a review of medical records. Demographic and clinical characteristics, biological test results and disease history were recorded by one physician (AD) with experience in managing patients with PMR. The disease manifestations of PMR—including weight loss (≥5%), morning stiffness (≥30 min), scapular and pelvic girdle arthromyalgia, and peripheral joint involvement (synovitis and/or tenosynovitis)—were evaluated. The following information was also collected for each patient: type of comorbidity (hypertension, dyslipidemia, and diabetes mellitus), history of any neoplasia, laboratory values, including C-reactive protein (CRP) and erythrocyte sedimentation rate (ESR), actual usage of corticosteroids and non-steroidal anti-inflammatory drugs (NSAIDs), and actual usage of methotrexate and biological disease-modifying anti-rheumatic drugs (bDMARDs).

#### 2.2.2. FDG-PET/CT Equipment and Protocol

Most of the FDG-PET/CT scans were performed in the participating hospitals’ nuclear medicine centers using the following scanners: (1) Siemens Biograph™ mCT (Siemens Healthinners) (Salengro Hospital, Lille University Hospital); (2) Discovery™ Rx (General Electrics Healthcare) (Huriez Hospital, Lille University Hospital); (3) Elite 710 (General Electrics Healthcare) (Valenciennes Hospital); and (4) Discovery™-690 (General Electrics Healthcare) (Oscar Lambret Center). Patients had to be fasting for 6 h, with a blood sugar level below 2 g/L. A dose of about 3 to 3.7 Mbq/kg of 18F-FDG was injected peripherally. CT acquisition began one hour after the injection, followed by PET acquisition. FDG-PET/CT images were interpreted using SyngoVia^®^ software (Siemens Healthcare).

In each patient and control, seventeen different articular and peri-articular sites were analyzed, including 2 glenohumeral joints, 2 acromio-clavicular joints, 2 sternoclavicular joints, 2 greater trochanters, 2 hips, 2 ischial tuberosities, 2 iliopectineal bursae, 2 pubic symphysis entheses, and the most inflammatory interspinous bursa (Supplementary Figures S1 and S2), as described by Sondag et al. [11].

PET images were available for all patients and were assessed visually by one independent specialist in nuclear medicine (FH), who was blinded to all patient and control data.

FDG-PET uptake was scored using a four-point scoring system, in which 0 = no FDG uptake; 1 = moderate FDG uptake, less than liver uptake; 2 = intense FDG uptake, equal to liver uptake; and 3 = intense FDG uptake, greater than liver uptake. For symmetrical skeletal regions (all but the interspinous bursa), scores from both sides were summed and divided by two. For each skeletal region, summed scores < 2 were considered to be negative, while scores ≥ 2 were considered positive. For each patient, we calculated a total skeletal score (TSS) by summing the scores at the 17 different skeletal sites (0–51) and the number of sites with FDG uptake ≥ 2 (0–17).

Additionally, in patients with isolated PMR [12]—symptoms typical of cranial GCA (visual problems, headache, or jaw pain) had to be absent—and their controls, PET scans were scored at thirteen different vascular regions (ascending thoracic aorta, aortic arch, descending thoracic aorta, abdominal aorta, pulmonary arteries, subclavian arteries, axillary arteries, vertebral arteries, and carotid arteries) using the same visual four-point scoring system (0, 1, 2, and 3) as described by Slart et al. [18]. For each of these patients, we calculated a total vascular score (TVS) by summing the individual scores at the 13 different vascular sites (0–39) and the number of sites with FDG uptake ≥ 2 (0–13). We made the choice to evaluate patients who were considered to have isolated PMR in order to assess silent underlying LVV which does not affect the temporal arteries, a disease that is not well recognized. 

### 2.3. Statistical Analysis

Categorical variables were expressed as numbers (%). Quantitative variables were expressed as means (standard deviation, SD) for normally distributed continuous variables or medians (interquartile range) for non-normally distributed continuous variables or semi-quantitative scores. The normality of distributions was assessed using histograms and the Shapiro–Wilk test.

Between-group comparisons (case vs. control) were performed using generalized linear mixed models (binomial distribution, logit function) for binary outcomes and linear mixed models for quantitative outcomes, with matched sets as a random effect. The normality of linear mixed model residuals was assessed and no deviations from normality were found. ROC curve analysis, based on the area under the ROC curve (AUC), was used to assess the ability of the number of sites with score ≥ 2 and the FDG uptake score to differentiate between the two groups. From the ROC curves, we determined optimal threshold values by maximizing the Youden index. Sensitivity and specificity for the optimal threshold were calculated. 

PMR patients were stratified according to use of corticosteroids and comparisons were performed using the Chi-square test for sex and the Student’s *t*-test or Mann–Whitney U test (regarding the normality of distributions) for quantitative variables.

Statistical testing was performed at the two-tailed α level of 0.05. Data were analyzed using the SAS software package, version 9.4 (SAS Institute, Cary, NC, USA).

## 3. Results

### 3.1. Patients and Controls

In the study period (from January 2015 to December 2019), 86 patients with a diagnosis of PMR had at least one FDG-PET/CT scan. Images were unavailable for four of the patients and uninterpretable for one patient (due to hyperinsulinism). 

Eighty-one patients with a diagnosis of PMR (mean (SD) age = 70.8 (9.8) years; 44.4% women) (Table 1) and 81 controls were included. Two-thirds of the patients had a diagnosis of PMR based on the EULAR/ACR 2012 criteria. For the rest of the patients, their diagnoses were based on expert opinions regarding clinical manifestations and imaging and were retained throughout the follow-up.

The patients were hospitalized either for diagnoses (n = 60) or reassessments of active PMR (n = 21). The choice to conduct FDG-PET/CT was made by the attending physician. Treatment resistance (22%), LVV (11%), and neoplasia (30%) were the three most common indications that were screened for. The indications of the FDG-PET/CT were unknown in 20 patients (25%).

Constitutional manifestations, such as weight loss and fever, were found in 40 patients (49.4%) and peripheral joint involvements were found in 22 patients (27.9%). The groups were comparable with regards to risk factors for cardiovascular disease (Table 1). A history of neoplasia was found in 20 patients (24.7%) with prostate cancers (n = 6), gastrointestinal cancers (n = 4), hematologic cancers (n = 4), breast cancers (n = 3), and others (n = 3).

The mean (SD) CRP was 60 mg/L (53). Forty-nine patients had been receiving concomitant treatments: four patients (4.9%) were being treated with Methotrexate (MTX), and forty-five patients (55.6%) were being treated with corticosteroids (mean (SD) dose of 15.3 (10.8) mg/day). None of the patients were on bDMARDs, such as tocilizumab. One case of PMR occurring after the use of an inhibitor of tyrosine kinase activity (bosutinib) was found in a patient with chronic myeloid leukemia. However, bosutinib is not a check point inhibitor and PMR was not a paraneoplastic condition in any case.

Ten patients had symptoms typical of cranial GCA and were not included in the LVV analysis. In those ten patients, a temporal artery biopsy was performed in nine of the patients, and, in all cases but one (result unavailable), the results were negative.

### 3.2. Comparison of FDG Uptake Score and Number of Sites with Score ≥ 2

Significant differences were found between the PMR and control groups at all sites (articular and periarticular) for the following: (i) the FDG uptake score (*p* < 0.001 for all locations); (ii) the number of patients per site with significant FDG uptake (score ≥ 2); (iii) the global FDG articular uptake scores (TSS, score of 0–51) (31 [IQR, 21 to 37] versus 6 [IQR, 3 to 10], *p* < 0.001); and (iv) the number of sites with significant FDG uptake (score ≥ 2) (score of 0–17) (11 [IQR, 7 to 13] versus 1 [IQR, 0 to 2], *p* < 0.001). Table 2 shows the global FDG articular uptake scores (TSS) and the number of sites with significant uptake (≥2) for the patients compared to the controls. Table 3 shows a site-by-site analysis.

### 3.3. Sensitivity and Specificity of FDG-PET/CT Findings for the Differential Diagnosis of PMR Compared to Controls

**Figure 1:**
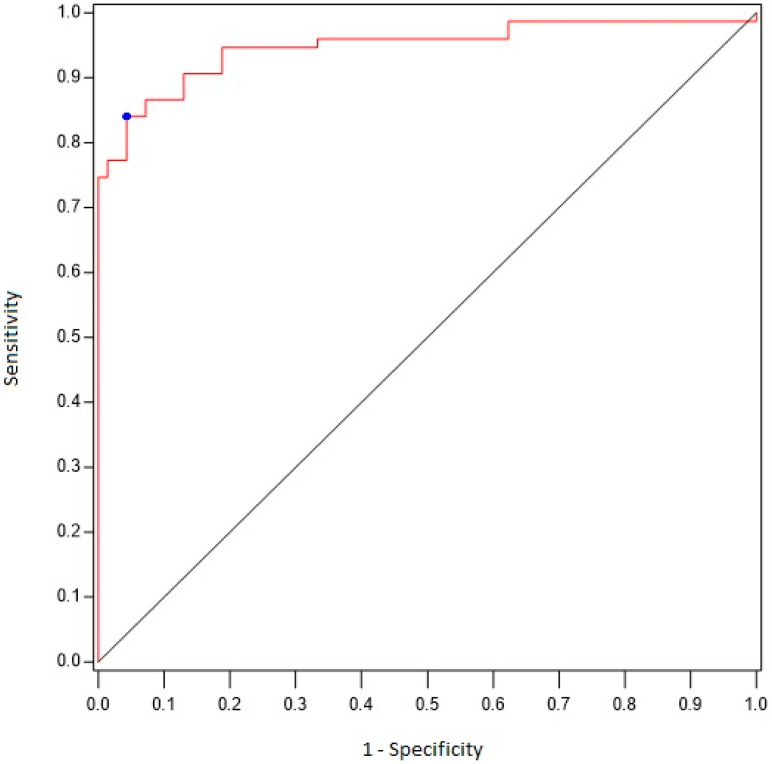
ROC curve analyzing the performance of FDG-PET/CT (number of sites) for the diagnosis of PMR.

Using ROC curve analyses (Figure 1), we found that the existence of six or more sites with scores ≥ 2 was associated with a diagnosis of PMR with 84% sensitivity and 96% specificity (AUC 0.90 [95% CI 0.85–0.95]). When a site-by-site analysis was performed (Table 4), we found that an uptake score ≥ 2 at the hips correlated with a diagnosis of PMR with 77% sensitivity and 96% specificity, whereas a score ≥ 2 at the shoulders correlated with a diagnosis of PMR with 81% sensitivity and 89% specificity.

### 3.4. Subgroup Analysis of the Effect of Corticosteroids on FDG Uptake

The PMR group was divided into two subgroups based on current corticosteroid use. There were 45 patients in the corticosteroids subgroup and 36 patients in the non-corticosteroids subgroup. The two subgroups were comparable in age (71.7 (10) years versus 69.6 (9.5); *p* = 0.35) and gender (48.9% men versus 63.9%; *p* = 0.18). Patients in the corticosteroids subgroup were hospitalized either for diagnoses (n = 35) or reassessments of active PMR (n = 10). The FDG uptake TSS and the number of sites with a score ≥ 2 were significantly higher in the non-corticosteroids subgroup compared to the corticosteroids subgroup: 35.5 [IQR, 31 to 42] versus 25 [IQR, 17 to 31] (*p* < 0.001) and 13 [IQR, 10 to 15] versus 9 [IQR, 6 to 11] (*p* < 0.001) respectively.

### 3.5. Comparison of FDG Vascular Uptake Score and Number of Patients with Score ≥ 2

For comparisons, 71 patients with isolated PMR (without any cranial ischemic manifestations of GCA) were matched 1:1 to controls based on age and gender. The patients were hospitalized either for diagnoses (n = 51) or reassessments of active PMR (n = 20). The controls were hospitalized either for ischemic strokes (n = 63) or hemorrhagic strokes (n = 8). No significant differences in global FDG vascular uptake scores (score 0–39) or in the number of patients with at least one significant uptake vascular site (score ≥ 2) were found between the PMR and control groups (1 [IQR, 0 to 4] versus 4 [0 to 6], *p* = 0.06 and 8 (11.3%) versus 10 (14.1%), *p* = 0.62 respectively) (Table 5). Only one patient with isolated PMR presented a classical pattern of extracranial GCA (Supplementary Figure S3). Nonetheless, this patient was already on corticosteroids. The choice to conduct FDG-PET/CT was made by the attending physician due to corticosteroids resistance and for screening for LVV.

The median FDG vascular uptake score (0–39) was higher in the non-corticosteroids group (n = 31) than in the corticosteroids group (n = 40, mean (SD) dose of 11.8 (6.7) mg/day), but without achieving statistical significance: 2 [IQR, 0 to 4] versus 0 [IQR, 0 to 4] (*p* = 0.53).

Moreover, in the 10 non-included patients with typical symptoms of cranial GCA, none exhibited significant vascular uptake.

## 4. Discussion

This multicenter, retrospective study included 81 patients with PMR and 81 controls and was conducted over a period of five years. The results show that the FDG uptake score and the number of sites with significant FDG uptake could be pertinent criteria for diagnosing PMR against a control group. However, unlike other authors, we did not confirm the presence of vascular involvement in patients with isolated PMR.

Our results are in accordance with those in the literature on the demographics (age and gender), clinical characteristics, and laboratory values of patients with PMR [11,12,15]. The frequency of having a history of cancer (one-fourth of the patients) was higher than expected. However, PMR was not a paraneoplastic condition in any case. In contrast with most previous studies, the patients with PMR were matched to controls (who had been hospitalized for stroke) for comparison, to visualize articular and periarticular FDG uptake as well as FDG vascular uptake at different locations. Furthermore, our control group is representative of the PMR population in terms of age, gender, and cardiovascular risk factors.

For the purpose of this study, we assessed FDG uptake at seventeen articular and periarticular regions using a TSS based on a four-point scoring system, as validated by Sondag et al. [11]. This TSS discriminates very well between PMR and non-PMR patients, whose TSS scores were 31 [21–37] and 6 [3–10], respectively. The number of sites with significant uptake also properly differs between PMR and non-PMR patients, with an optimal cut-off value of 6 (84% sensitivity and 96% specificity). A somewhat similar approach was adopted by several other authors [11,15,19]. Sondag et al. analyzed seventeen hotspots on FDG-PET/CT scans completed on 50 patients with a diagnosis of PMR and 53 patients with a neoplasm as a control group [11]. The presence of ≥three sites with significant uptake correlated with a diagnosis of PMR, with 74% sensitivity and 79% specificity [11]. Henckaerts et al. prospectively included 99 patients with a plausible diagnosis of PMR. All patients underwent PET scans before commencing corticosteroids [15]. FDG uptake was scored for twelve articular regions (scores 0–2) and a TSS was calculated (total of 0–24) reflecting the FDG uptake in those 12 articular regions. Of the 99 patients, 67 were diagnosed with PMR, while another diagnosis was established for the remaining 32 patients based on the judgment of an experienced clinician. For PMR, a TSS of >16 had a sensitivity of 85.1% and specificity of 87.5% [15]. Takahashi retrospectively analyzed the PET scans of ten patients with elderly onset rheumatoid arthritis (RA) and twenty-seven patients with PMR [19]. The FDG uptake in great joints (such as hips, shoulders, elbows, and wrists) and in bursal sites (such as greater trochanters, spinous processes, and ischial tuberosities) was assessed. The FDG-PET uptake was scored using a five-point scoring system with 0 indicating no uptake (same as bone), and 4 as the highest uptake (SUV > 4). An FDG uptake score ≥ 2 was considered significant. The authors identified five characteristics with high sensitivity and specificity for distinguishing PMR from elderly onset RA: (i) uptake at the ischial tuberosities, (ii) uptake at the spinous processes, (iii) non-appearance of uptake at the wrists, (iv) single uptake at the iliopectineal bursa, and (v) non-appearance of uptake at the shoulders. When at least three of these characteristics were present, the sensitivity and specificity were 92.6% and 90.0%, respectively [19]. In our study, the sites with the highest specificity were the shoulders and the ischial tuberosities, which has already been observed in other studies [11,19]. Our results support the utility of FDG-PET/CT in the assessment of PMR lesions. Furthermore, our sensitivity and specificity estimates were higher than those reported in previous studies, and our study is one of the larger-scale case-control studies that makes use of powerful semi-quantitative scoring.

A great challenge for doctors who treat PMR is identifying patients with silent underlying LVV. Intriguingly, a positive FDG-PET/CT scan demonstrating the presence of LVV is seen in between 7% and 60.7% of cases, depending on the target population, the acquisition protocol, and the criteria used. In a study conducted by Blockmans et al., including thirty-five patients with isolated PMR, vascular FDG uptake was observed in eleven participants (31%), predominantly at the subclavian arteries [12]. The vascular FDG uptake was scored at seven different vascular sites using a four-point scoring system, and only a score of 1 was required, which may explain the high percentage of positivity [12]. Henckaerts et al. observed a vascular FDG uptake score of 2 in at least one large vessel in 15% of PMR patients (n = 67) and 6% of non-PMR patients (n = 32, seronegative RA (n = 3), osteoarthritis (n = 5), rotator cuff pathology (n = 8), and spontaneous resolution (n = 8)) (not significantly different) [15]. In that study, FDG-PET/CT scans were scored at four different vascular sites (carotid arteries, subclavian arteries, thoracic aorta, and abdominal aorta) using a visual three-point scoring system (0, 1, and 2) [15]. Prieto-Peña et al. found a positive FDG-PET/CT scan (score ≥ 2) demonstrating the presence of LVV in 60.7% of patients with isolated symptoms of PMR [20]. In that study, FDG-PET/CT scans were scored at five different vascular sites (femoral/tibio-peroneal arteries, iliac arteries, abdominal aorta, thoracic aorta, and supra-aortic trunks) [20]. Our finding that only 11.3% of the PMR patients exhibited an increase in vascular FDG uptake, compared with 14.1% of the control patients, may seem at odds with the 60.7% of PMR patients with vascular FDG uptake reported by Prieto-Peña et al. [20]. However, the high percentage of LVV reported by Prieto-Peña et al. might be due partly to the criteria used for the analysis of images, since they used an acquisition protocol with a longer delay than is generally used in practice [20]. More importantly, we were able to compare our findings with a representative control group. Our results could be explained in part by the following: (i) the presence of large-vessel atherosclerosis, since the FDG-PET/CT scans were performed on controls who had suffered a stroke; (ii) as a result of selecting patients with “pure” PMR or a lack of selection of patients with atypical manifestations of the disease. However, the most important factor for determining whether there is a silent underlying LVV is probably the pattern of significant vascular uptake rather than the global FDG vascular uptake score. Camellino et al. [21] recently found that a positive FDG-PET/CT scan (score ≥ 2) demonstrates the presence of LVV in 11.9% of patients with isolated symptoms of PMR, which is similar to our findings.

The strengths of this study include the following: (1) the large number of patients and its multicentric design (patients from two centers in the Hauts-de-France region of France were included); (2) the fact that all the medical records were systematically assessed by a single investigator (AD) with experience in managing patients with PMR; (3) the fact that all FDG-PET/CT images were thoroughly evaluated visually by a single independent specialist in nuclear medicine (FH) who was blinded to all patient and control data; and (4) the results regarding LVV detection probably approach real life better than those from initial studies because we included an acquisition time of 60 min after the 18FDG injection whereas other authors have suggested a more delayed time for vascular imaging. We admit several limitations, including the retrospective nature of this study and the fact that the PMR diagnoses were based on expert opinions rather than the 2012 EULAR/ACR classification criteria. We also included patients on corticosteroids. In these patients, the FDG uptake score and the number of sites with significant uptake were significantly lower than in the non-corticosteroids group. The fact that the control group comprised non-rheumatic patients is another limitation. FDG-PET/CT scans were performed in the participating hospitals’ nuclear medicine centers using different types of equipment. However, the PET protocol acquisition was similar in each center and the FDG-PET/CT images were interpreted by one independent specialist in nuclear medicine using SyngoVia^®^ software (Siemens Healthcare). Finally, our results cannot be extrapolated to the general population of patients with PMR because the patients in our study were hospitalized whereas typical patients with PMR are managed as outpatients, which shows added severity or diagnostic concerns. This is illustrated by the high number of patients with glucocorticoid resistance reported (22%).

The use of FDG-PET/CT in every suspected case of PMR should not be recommended. In patients with typical clinical presentations, corticosteroids can be commenced based on the combination of plausible clinical symptoms and serologic markers of inflammation alone. If the clinical presentation exhibits some atypical features, if the serologic markers of inflammation are not as high as expected, or if there is a history of cancer, FDG-PET may be a reliable aid. Based on our findings, the use of FDG-PET in every patient with isolated PMR is not suggested to identify individuals with silent underlying LVV. However, FDG-PET could be a useful aid in (i) the assessment of underlying LVV in patients with corticosteroid-resistant PMR without cranial ischemic manifestations [22,23] and (ii) in the early diagnosis of underlying extracranial LVV in PMR patients with constitutional symptoms and/or with ischemic extracranial manifestations, mainly with upper- or lower-limb ischemia [23]. Moreover, PET is a medical tool used in clinical settings and its high initial cost is no longer a limitation since PET imaging costs have decreased dramatically over the years. However, the costs may be high, depending on the country.

In conclusion, over a period of five years, a large number of patients with PMR benefited from FDG-PET/CT imaging, even though there is no recommendation for its use. The inclusion of FDG-PET/CT in a step-by-step diagram for the classification and diagnosis of PMR needs further evaluation in the future.

## 5. Significance and Innovation

- FDG-PET/CT might be an important diagnostic tool in patients with suspected polymyalgia rheumatica;
- No significant differences in the global FDG vascular uptake scores were found between the patients who were considered to have isolated PMR and the control groups.

## Figures and Tables

**Table 1:** Clinical characteristics of patients.

	PMR Group		Control Group	
	N	Mean ± SD Median [IQR] or Number (%)	N	Mean ± SD Median [IQR] or Number (%)
Age at inclusion, years	81	70.8 ± 9.7 71.0 [62.9; 79.1]	81	70.7 ± 9.9 71.0 [61.7; 78.4]
Female sex	81	37 (45.7)	81	37 (45.7)
Body Mass Index, kg/m^2^	64	26.9 ± 4.8 26.5 [23.3; 30.1]	40	25.5 ± 6.1 24.9 [21.9; 27.2]
Hypertension	77	54 (70.1)	81	55 (67.9)
Dyslipidemia	80	33 (41.3)	81	43 (53.1)
Diabetes mellitus	81	17 (21.0)	81	23 (28.4)
History of neoplasia	81	20 (24.7)	81	13 (16.0)
Clinical characteristics				
Fever (temperature ≥ 38 °C)	74	7 (9.5)	-	-
Weight loss (≥5%)	67	32 (47.8)	-	-
Morning stiffness (≥30 min)	52	40 (76.9)	-	-
Shoulder girdle pain	79	69 (87.3)	-	-
Hip girdle pain	79	45 (57.0)	-	-
Peripheral joint involvement	79	22 (27.9)	-	-
Laboratory values				
ESR, mm/h	61	42 ± 28 39 [24; 52]	-	-
CRP, mg/L	81	60 ± 53 42 [15; 102]	-	-
Treatment				
Current corticosteroids	81	45 (55.6)	-	-
Corticosteroids dose	45	15.3 ± 10.8 14.0 [6.8; 20.0]	-	-
Current NSAID	81	22 (27.2)	-	-
Current MTX	81	4 (4.9)	-	-

SD: Standard deviation; IQR: Interquartile range; kg/m^2^: kilogram per square meter; °C: Degrees Celsius; min: Minutes; ESR: Erythrocyte sedimentation rate; CRP: C-reactive protein; NSAID: Nonsteroidal anti-inflammatory drug; MTX: Methotrexate.

**Table 2:** FDG-PET/CT results in patients and controls.

	PMR Group (n = 81)	Control Group (n = 81)	*p*-Value
Total skeletal score (0–51), FDG uptake (0–3) for each site	31 [21 to 37]	6 [3 to 10]	<0.001
Number of sites (0–17) with significant uptake (≥2)	11 [7 to 13]	1 [0 to 2]	<0.001

Values are expressed as median [interquartile range].

**Table 3:** FDG-PET/CT results according to location.

Location *	FDG Uptake Score (0–3)			Patients with Significant Uptake (≥2)		
	PMR Group (n = 81)	Control Group (n = 81)	*p*-Value	PMR Group (n = 81)	Control Group (n = 81)	*p*-Value
Acromioclavicular joints	1.5 [1 to 2]	1 [0 to 1]	<0.001	31 (38.3)	6 (7.4)	<0.001
Glenohumeral joints	2.5 [2 to 3]	1 [0 to 1.5]	<0.001	65 (81.3)	9 (11.1)	<0.001
Sternoclavicular joints	1 [0 to 2.5]	0 [0 to 0]	<0.001	31 (38.3)	0 (0)	NA
Greater trochanters	2 [1 to 2.5]	1 [0.5 to 1]	<0.001	51 (63.0)	6 (7.4)	<0.001
Hips	3 [2 to 3]	0 [0 to 1]	<0.001	58 (77.3)	3 (4.3)	<0.001
Ischial tuberosities	2 [1 to 3]	0 [0 to 0]	<0.001	55 (67.9)	2 (2.5)	<0.001
Iliopectineal bursae	1 [0 to 2]	0 [0 to 0]	<0.001	23 (30.7)	0 (0)	NA
Pubic symphysis entheses	0 [0 to 1.5]	0 [0 to 0]	<0.001	19 (23.5)	1 (1.2)	0.003
Interspinous process	2 [0 to 3]	0 [0 to 1]	<0.001	48 (59.3)	7 (8.6)	<0.001

Values are expressed as median [interquartile range] or numbers (percentage). NA: not applicable. * For symmetrical skeletal regions (all but interspinous process), scores from both sides were summed and divided by two.

**Table 4:** Optimal cut-off values for FDG uptake score.

Location *	Area under the Curve **	Optimal Cut-Off Values for FDG Uptake Score	Sensitivity	Specificity
Acromioclavicular joints	0.73	1.5	0.56	0.77
Glenohumeral joints	0.89	2	0.81	0.89
Sternoclavicular joints	0.80	0.5	0.72	0.81
Greater trochanters	0.84	1.5	0.74	0.83
Hips	0.92	2	0.77	0.96
Ischial tuberosities	0.89	1	0.84	0.83
Iliopectineal bursae	0.81	0.5	0.64	0.96
Pubic symphysis entheses	0.71	0.5	0.49	0.90
Interspinous process	0.78	2	0.59	0.91

* For symmetrical skeletal regions (all but interspinous process), scores from both sides were summed and divided by two. ** Area under the curve were calculated for FDG uptake score treated as continuous.

**Table 5:** FDG-PET/CT results according to location (vascular uptake).

	FDG Uptake Score (0–3)		Patients with Significant Uptake (≥2)	
	PMR Group (n = 71)	Control Group (n = 71)	PMR Group (n = 71)	Control Group (n = 71)
Ascending thoracic aorta	0 [0 to 0]	0 [0 to 0]	0 (0)	0 (0)
Aortic arch	0 [0 to 0]	0 [0 to 0]	0 (0)	0 (0)
Descending thoracic aorta	0 [0 to 0]	0 [0 to 0]	1 (1.4)	0 (0)
Abdominal aorta	0 [0 to 0]	1 [0 to 0]	1 (1.4)	0 (0)
Pulmonary arteries	0 [0 to 0]	0 [0 to 0]	0 (0)	0 (0)
Subclavian arteries	0 [0 to 1]	0 [0 to 1]	3 (4.2)	7 (9.8)
Axillary arteries	0 [0 to 1]	1 [0 to 1]	2 (2.8)	4 (5.6)
Vertebral arteries	0 [0 to 0]	0 [0 to 0]	1 (1.4)	0 (0)
Carotid arteries	0 [0 to 0]	0 [0 to 0]	0 (0)	1 (1.4)

Values are expressed as median [interquartile range] or numbers (percentage). NA: not applicable. For symmetrical vascular regions (subclavian arteries, axillary arteries, vertebral arteries, carotid arteries), scores from both sides were summed and divided by two.

## Data Availability

The data presented in this study are available on request from the corresponding author.

## Supplementary Materials

The following supporting information can be downloaded at: https://www.mdpi.com/article/10.3390/jcm12082844/s1, Figure S1: Total Skeletal Score (0–51); Figure S2: FDG uptake at the hips (A and C), shoulders (B and C) and lumbar interspinous bursa (D) in a 68-year-old woman with a Total Skeletal Score of 47/51; Figure S3: FDG uptake at the axillary arteries (A), aortic arch (A), subclavian arteries (B) and left vertebral artery (C) in a 73-year-old woman with a Total Vascular Score of 25/39.
