# Supplementary material for: Usefulness of 18F-Fluorodeoxyglucose Positron Emission Tomography in Diagnosing Polymyalgia Rheumatica and Large-Vessel Vasculitis: A Case-Control Study
Source: J Clin Med. 2023 Apr 13;12(8):2844. doi: 10.3390/jcm12082844 (PMC10141365; doi:10.3390/jcm12082844)

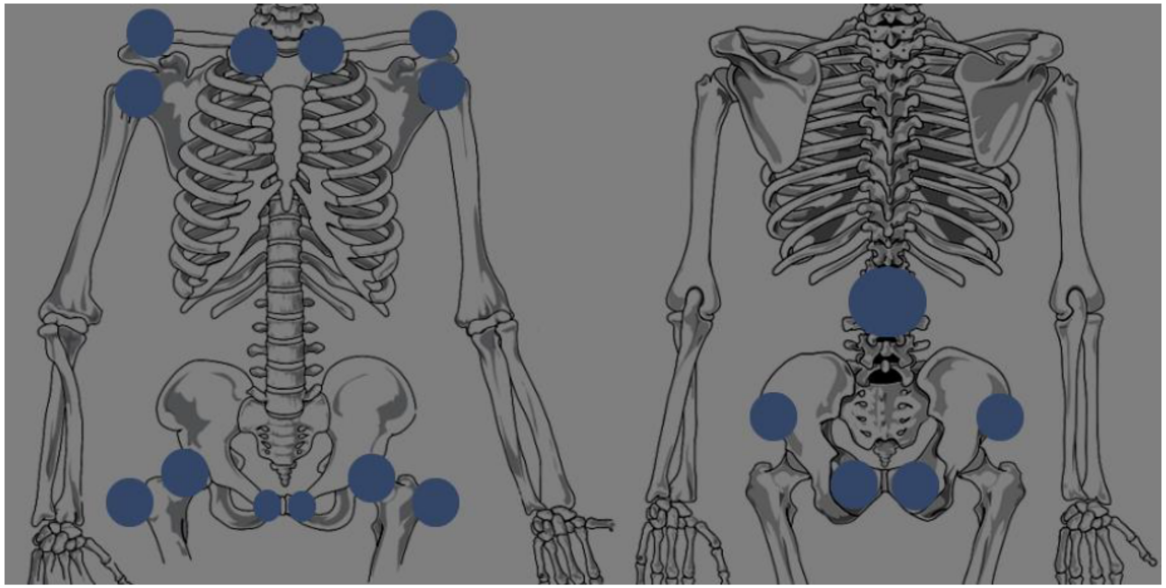

Supplementary Figure S1: Total Skeletal Score (0-51)
FDG uptake was scored visually for 17 articular or periarticular sites using a semi-quantitative scoring system (score 0-3), as described by Sondag et al. [11]. Scores were established for two glenohumeral joints, two acromio-clavicular joints, two sternoclavicular joints, two greater trochanters, two hips, two ischial tuberosities, two iliopectineal bursae, two pubic symphysis entheses and the most inflammatory interspinous bursa. The individual scores at the 17 different sites were summed to obtain the total skeletal score (0-51).

Supplementary Figure S2: FDG uptake at greater trochanter (A and C), shoulders (B and C), and lumbar interspinous bursa (D)

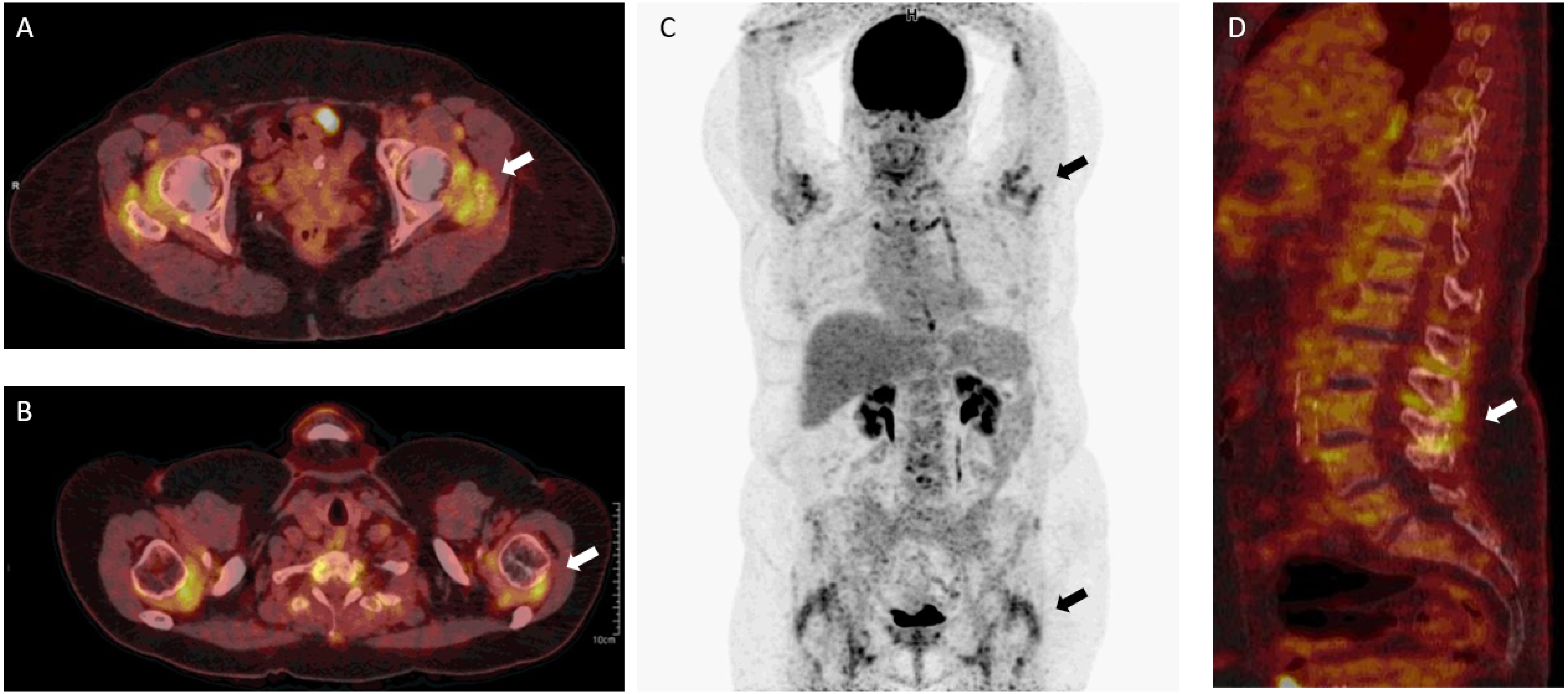

Supplementary Figure S3: FDG uptake at the axillary arteries (A), aortic arch (A), subclavian arteries (B) and left vertebral artery (C) in a 73-year-old woman with a total vascular score of 25/39

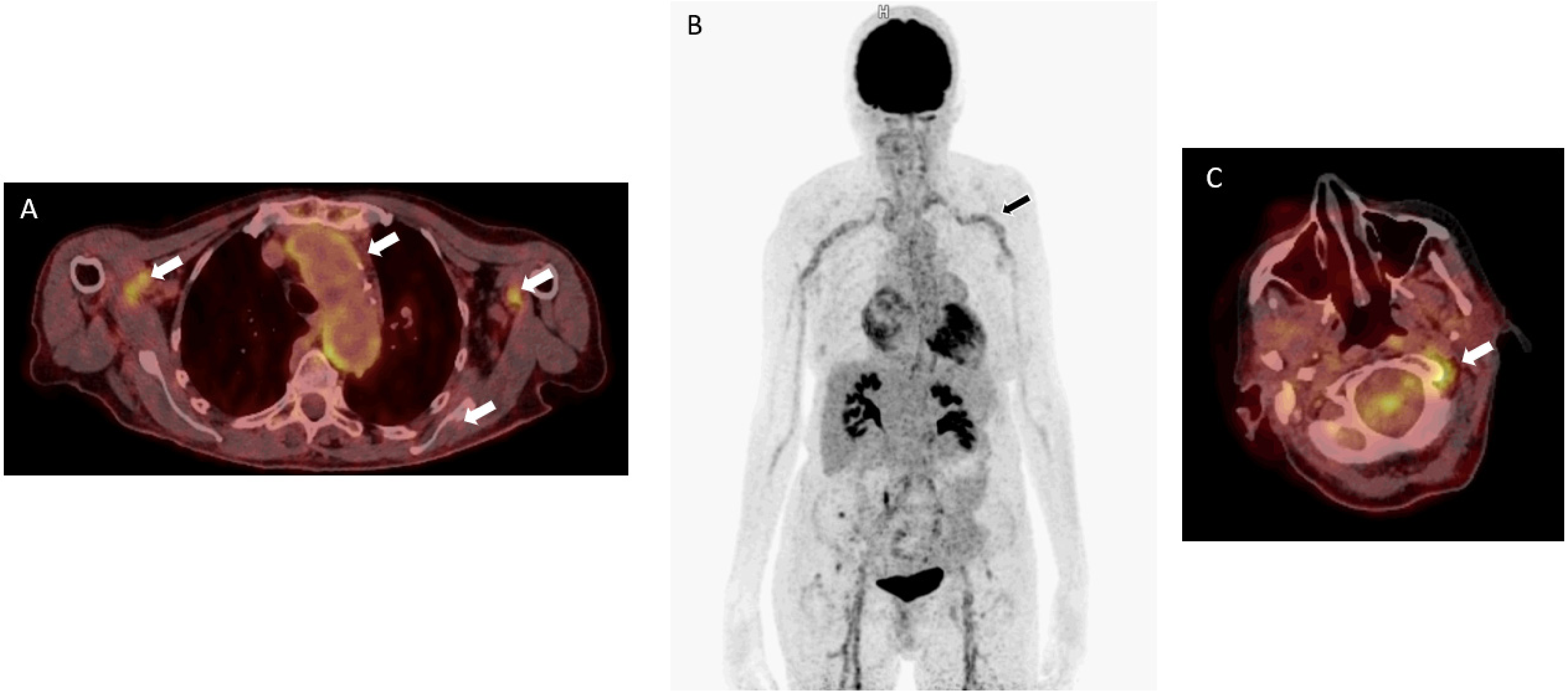

Supplement: Supplementary file 1 [file jcm-12-02844-s001.zip › jcm-2291500-supplementary.pdf]
